# Failure Locus of an ABS-Based Compound Manufactured through Photopolymerization

**DOI:** 10.3390/polym14183822

**Published:** 2022-09-13

**Authors:** Dan-Andrei Șerban, Alexandru Viorel Coșa, George Belgiu, Radu Negru

**Affiliations:** 1 Department of Mechatronics, Politehnica University Timişoara, 300222 Timişoara, Romania; 2 Department of Management, Politehnica University Timişoara, 300191 Timişoara, Romania; 3 Department of Mechanics and Strength of Materials, Politehnica University Timişoara, 300222 Timişoara, Romania

**Keywords:** failure, ductile damage, photopolymerization, mechanical testing, numerical analyses

## Abstract

This work investigates the critical plastic strain variation with stress triaxiality and the Lode angle parameter for an Acrylonitrile butadiene styrene (ABS)-based proprietary blend compound (commercial name VeroWhitePlus™ RGD835) manufactured through photopolymerization. Various triaxial states of stress and Lode angles were obtained with the help of notched flat specimens used in tensile loadings, notched round specimens used in compression (upsetting) tests and butterfly specimens used in Arcan tests. The tests were replicated using finite element analyses in order to evaluate the aforementioned parameters.

## 1. Introduction

The evaluation of the degradation and failure of materials and structures has been an important research topic in the past century, due to the ever-growing need of precise dimensioning of load-bearing structures. The first studies regarding structural integrity laid the foundations of fracture mechanics and were focused on the nucleation and propagation of cracks, as well as the conditions in which these types of defects can lead to a premature failure of a component [1,2,3,4]. Though very successful in the lifetime prediction of components subjected to fatigue loadings or the case of structural defects that cause high degrees of stress concentration (i.e., crack propagation), the principles of fracture mechanics cannot be implemented in static or dynamic applications where the failure was caused by loads that exceed the strength of the material.

In contrast to the microscopic approach used in fracture mechanics, Nobel laureate in Physics Percy Williams Bridgman proposed a phenomenological failure model for ductile materials based on the equivalent plastic strain ${\bar{\varepsilon }}^{pl}$ and the stress triaxiality η in the critical region [5]. The concept behind this approach is that, at the onset of damage, the local strain increases drastically when compared to the global strain of the body (an example of this phenomenon is the necking observed in tensile tests on ductile materials).

The equivalent plastic strain ${\bar{\varepsilon }}^{pl}$ is a scalar that can be determined based on the plastic strain rate tensor components ${\dot{\varepsilon }}_{ij}^{pl}$ and the equivalent stress $\bar{\sigma }$ defined by a given yield criterion, using the principle of energetic conjugates [6].

(1) $$\bar{\sigma }\cdot {\bar{\dot{\varepsilon }}}^{pl}=\overset{n}{\underset{i,j=1}{\sum }}\sigma_{ij}{\dot{\varepsilon }}_{ij}^{pl}$$

For the von Mises equivalent stress, the equivalent plastic strain ${\bar{\varepsilon }}^{pl}$ is based on the second invariant of the plastic strain tensor and has the relation

(2) $${\bar{\varepsilon }}^{pl}=\int {\bar{\dot{\varepsilon }}}^{pl}dt=\int \left(\sqrt{\frac{2}{3}{\dot{\varepsilon }}_{ij}^{pl}{\dot{\varepsilon }}_{ij}^{pl}}\right)dt$$

(3) $${\bar{\varepsilon }}^{pl}=\sqrt{\frac{2}{3}\varepsilon_{ij}^{pl}\varepsilon_{ij}^{pl}}$$

The stress triaxiality η is a scalar value based on the first invariant $I_{1}$ of the stress tensor σ and the second invariant $J_{2}$ of the deviatoric stress tensor σ′

(4) $$\eta =\frac{1}{3}\frac{I_{1}}{\sqrt{3J_{2}}}$$

(5) $$I_{1}=tr\left(\sigma \right)=\sigma_{1}+\sigma_{2}+\sigma_{3}$$

(6) $$J_{2}=\sigma \prime :\sigma \prime =\frac{1}{6}\left[{\left(\sigma_{1}-\sigma_{2}\right)}^{2}+{\left(\sigma_{2}-\sigma_{3}\right)}^{2}+{\left(\sigma_{3}-\sigma_{1}\right)}^{2}\right]$$

In engineering terms, the stress triaxiality can be expressed as the hydrostatic stress $\sigma_{Hid}$ divided by the von Mises equivalent stress $\sigma_{Mises}$.

(7) $$\eta =\frac{\sigma_{Hid}}{\sigma_{Mises}}=\frac{\sigma_{1}+\sigma_{2}+\sigma_{3}}{\frac{3}{\sqrt{2}}{\left[{\left(\sigma_{1}-\sigma_{2}\right)}^{2}+{\left(\sigma_{2}-\sigma_{3}\right)}^{2}+{\left(\sigma_{3}-\sigma_{1}\right)}^{2}\right]}^{\frac{1}{2}}}$$

(8) $$\sigma_{Hid}=\frac{\sigma_{1}+\sigma_{2}+\sigma_{3}}{3}$$

(9) $$\sigma_{Mises}=\sqrt{3J_{2}}=\frac{1}{\sqrt{2}}{\left[{\left(\sigma_{1}-\sigma_{2}\right)}^{2}+{\left(\sigma_{2}-\sigma_{3}\right)}^{2}+{\left(\sigma_{3}-\sigma_{1}\right)}^{2}\right]}^{\frac{1}{2}}$$

According to Bridgman’s hypothesis, the critical equivalent plastic strain (that determines the localized failure of the material) ${\bar{\varepsilon }}_{cr}^{pl}$ varies with the stress triaxiality for a given material. This principle was based on the experimental observation that, during loading, ductile metals exhibit nucleation of voids in their structure, that increase with straining, ultimately leading to the failure of the specimen. 

Based on experimental data gathered from several materials, Gordon R. Johnson and William H. Cook proposed an analytical function that describes the variation of the critical equivalent plastic strain ${\bar{\varepsilon }}_{cr}^{pl}$ with the stress triaxiality η, the equivalent plastic strain rate ${\bar{\dot{\varepsilon }}}^{pl}$ and the homogenous temperature $\bar{T}$ [7].

(10) $${\bar{\varepsilon }}_{cr}^{pl}\left(\eta ,{\bar{\dot{\varepsilon }}}^{pl},\bar{T}\right)=\left[D_{1}+D_{2}\cdot e^{D_{3}\cdot \eta }\right]\left[1+D_{4}\ln{\bar{\dot{\varepsilon }}}^{pl}\right]\left[1+D_{5}\bar{T}\right]$$

where $D_{1},D_{2},\;D_{3},\;D_{4}$ and $D_{5}$ are material constants. 

Further developments of the ductile damage criterion considered a more complete description of the stress state by taking into account the influenced by the third invariant of the deviatoric stress tensor $J_{3}$, through the Lode angle Θ, which represents the angular coordinate of the cylindrical frame of reference of the Haigh–Westergaard stress space [8].

(11) $$J_{3}=det\left(\sigma \prime \right)=\frac{27}{2}\left[\left(\sigma_{1}-\sigma_{Hid}\right)\left(\sigma_{2}-\sigma_{Hid}\right)\left(\sigma_{3}-\sigma_{Hid}\right)\right]$$

The current approach in dealing with the influence of the third invariant of the deviatoric stress tensor $J_{3}$ considers the Lode angle parameter ξ, defined as [9,10]

(12) $$\xi =cos\left(3\Theta \right)=\frac{3}{2}\sqrt{3}\frac{J_{3}}{J_{2}{}^{\frac{3}{2}}}=\frac{J_{3}}{{\left(\sigma_{Mises}\right)}^{3}}$$

The successful failure prediction of metals using the ductile failure models implemented in finite element analysis software is well documented in literature [10,11,12] and, in recent years, it has been applied to polymeric materials with good results [13,14], considering the limited model calibration data. 

Once the damage initiation criterion is achieved, the progressive damage and failure of the material can be modelled using a damage evolution function, that gradually diminishes the effective stress tensor components by considering various parameters (usually, equivalent plastic strain or strain energy) [14,15].

The aim of this study is to perform a series of experimental procedures for various loading conditions (tension, compression, shear) on specimens with special geometries, that can determine a wide range of variation for the stress triaxiality and the Lode angle parameter and eventually determine the variation of the critical plastic strain with the aforementioned parameters. Considering the fact that the machining of polymers can alter their mechanical behaviour in a significant manner [16] and that the development cost of specialized injection moulds for each geometry is costly, the evaluation of testing protocols designed for metals will be performed on rapid prototyped parts. All the specimens presented in this study were manufactured through photopolymerization, using the PolyJet technology [17,18,19]. The material used has the commercial name VeroWhitePlus™ RGD835, and represents an ABS based proprietary blend manufactured by Stratasys [20]. 

The experimental procedures consisted of tensile tests on flat notched specimens, upsetting tests on cylindrical and round notched specimens and Arcan tests, with five specimens being tested for each geometry/loading condition. A low dispersion of results was observed for each set of identical tests, the largest recorded standard deviations being 7% for the stiffness (Arcan tests at 15° orientation) and 9% for the recorded strength (upsetting tests for the specimens with a notch radius of 6.67 mm). Finite element analyses were used to replicate the experimental procedures and determine the variation of the investigated parameters in the critical region. Considering the low dispersion of results for all the tests performed, the most representative stress–strain curves for each specimen type/loading condition (ones exhibiting the stiffness and strength closest to the average values) were used as a benchmark for the comparison with the numerical data (hence, only one simulation was performed for each specimen type/loading condition).

## 2. Tensile Tests on Flat Specimens

**Figure 1:**
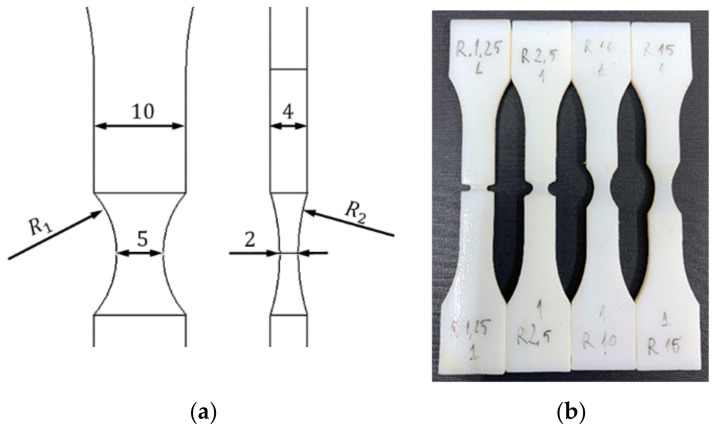
Details of the notch geometry (**a**) and the four types of manufactured specimens (**b**).

The tensile tests were performed on flat specimens that presented a transition area from the critical region to the body of the samples through double fillets of varying radii (Figure 1a). The geometry of the specimens was based on ISO 527 specifications [21], having a 4 mm × 10 mm cross-section area in the calibrated region. The critical region was kept identical for all specimen geometries (2 mm × 5 mm).

The values for the fillet radius $R_{1}$ was chosen based on the relation proposed by Bao for estimating the stress triaxiality η of notched specimens as a function of the minimum cross section width t (equalling 5 mm for this case) and the circumferential notch radius $R_{1}$ [11].

(13) $$\eta =\frac{1}{3}+\sqrt{2}ln\left(1+\frac{t}{2R_{1}}\right)$$

Considering the analytical estimation of the variation of the stress triaxiality η with circumferential notch radius, four values were chosen for $R_{1}$: 1.25 mm, 2.5 mm, 10 mm and 15 mm.

The values for the second fillet radii were chosen based on geometrical constraints for each specimens: three-point arcs were generated, intersecting the middle of the specimen (the critical region, with a distance of 1 mm from the mid plane of the specimen) and the surface of the calibrated region that resulted from the $R_{1}$ notch (Figure 1a).

The specimens were manufactured through photopolymerization, using the PolyJet technology, the procedure and parameters being detailed in [19], the resulting specimens being presented in Figure 1b.

**Figure 2:**
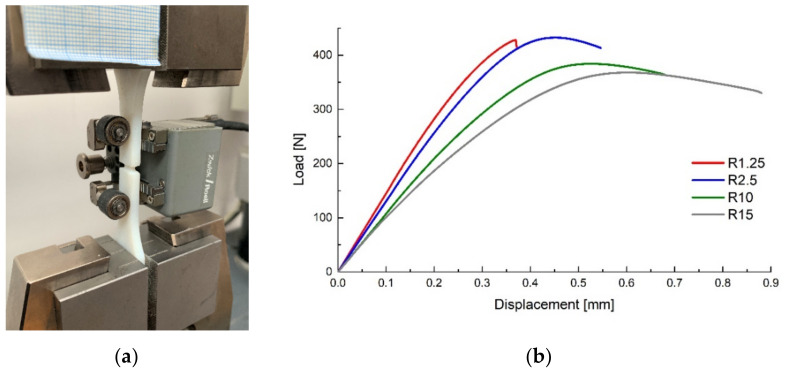
Experimental setup (**a**) and force–displacement curves for the tensile specimens (**b**).

The experimental procedures were carried out on a 5 kN Z005 universal testing machine manufactured by Zwick Roell (Ulm, Germany) at ambient temperature using a 1 mm min^−1^ crosshead travel speed. In order to develop accurate simulations, a strain-gauge extensometer was used to record the relative displacement of the surfaces neighbouring the critical region, as depicted in Figure 2a. Representative force–displacement curves for each specimen type are presented in Figure 2b, the higher stress concentration caused by the smaller notch radii determining stiffer responses and lower fracture strains. As was expected, all specimens failed in the critical region.

Finite element analyses were performed in the commercial software Abaqus/CAE 2019 (Dassault Systèmes, Vélizy-Villacoublay, France), using the implicit solver (Abaqus Standard) on models based on the geometries used for rapid prototyping, reduced to the region contained between the extensometer grips. The material formulation used consisted of an elastic-plastic formulation with multi-linear isotropic hardening (detailed in [19,22]). The bottom of the model was fixed while a different displacement was applied to the top of the model for each geometry, corresponding to the travel recorded by the extensometer at specimen failure. The models were meshed using second order tetrahedral elements (C3D10).

**Figure 3:**
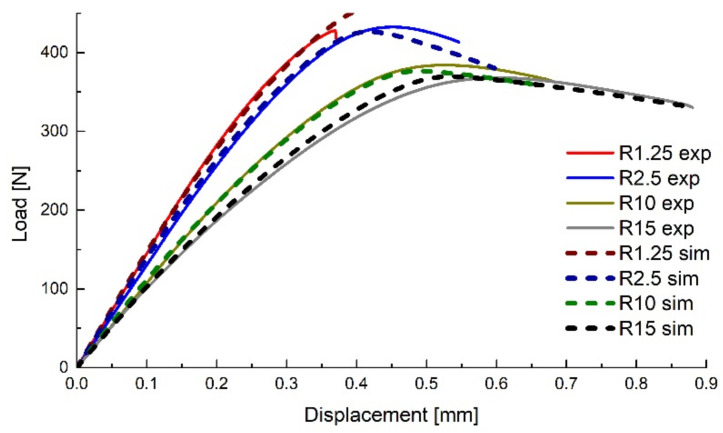
Comparison between the experimental and numerical force–displacement curves for the flat specimens.

The force–displacement curves resulted from the analyses are presented in Figure 3, showing a good correlation between the experimental and numerical data. 

**Figure 4:**
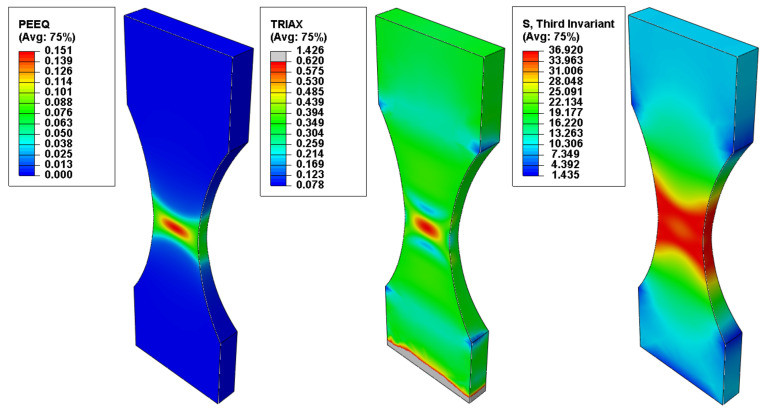
Equivalent plastic strain, stress triaxiality and normalized third invariant variation in the middle of the specimens for the 15 mm radius tensile tests.

**Figure 5:**
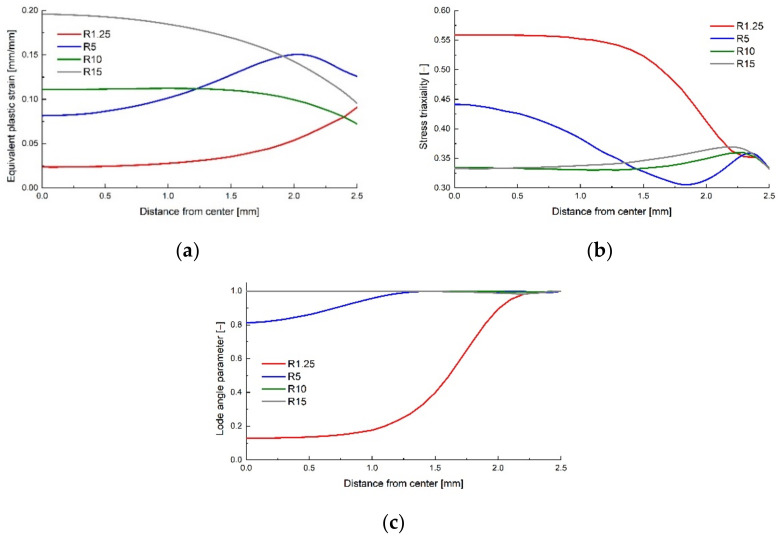
Equivalent plastic strain (**a**), stress triaxiality (**b**) and Lode angle parameter (**c**) variation with the distance from the centre of the specimen to the extremities.

The recalled field output variables of interest were the Von Mises equivalent stress $\sigma_{Mises}$, the stress triaxiality η, the normalized third invariant of the deviatoric stress tensor $\rho =\sqrt[{3}]{J_{3}}$ and the equivalent plastic strain ${\bar{\varepsilon }}^{pl}$ [23]. The Lode angle parameter was calculated using Equation (12). A path of integration points was defined in the mid plane of the specimens, ranging from their center to the extremity (Figure 4) in order to plot the variation of the equivalent plastic strain, stress triaxiality and Lode angle parameter with the distance from the center (Figure 5).

Considering the hypothesis that the nucleation of voids occurs in the middle of the specimen, the corresponding values for the equivalent critical plastic strain (recorded at the end of each simulation), stress triaxiality and Lode angle parameter are presented in Table 1. The values for the tensile test at 0.333 stress triaxiality were determined for the tensile tests performed on un-notched specimens, detailed in a previous study [19].

**Figure 6:**
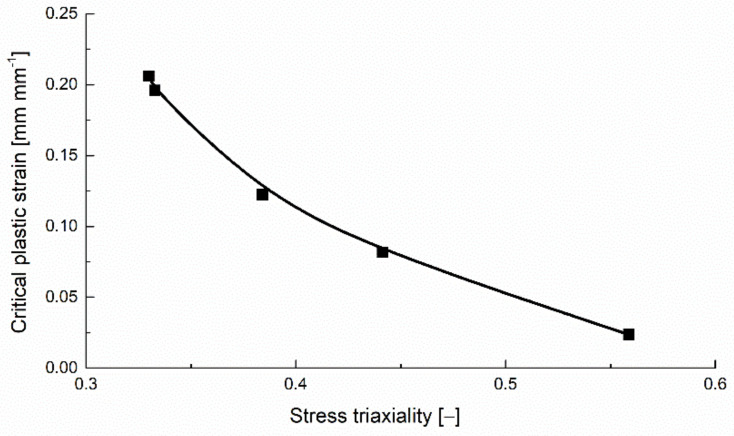
Critical plastic strain variation with stress triaxiality for the notched flat tensile specimens.

The variation of the critical equivalent plastic strain with the stress triaxiality is presented for the flat specimens in Figure 6.

## 3. Upsetting Tests

The values of the critical equivalent plastic strain for negative stress triaxialities were determined using upsetting tests on cylindrical specimens and round notched specimens with various notch radii r. The values of r were chosen with the help of Bridgman’s relation, that predicts the variation of the stress triaxiality with the notch radius and the minimal radius of the cylindrical specimens *a* = 4 mm [5].

(14) $$\eta =-\left[\frac{1}{3}+ln\left(1+\frac{a}{2r}\right)\right]$$

**Figure 7:**
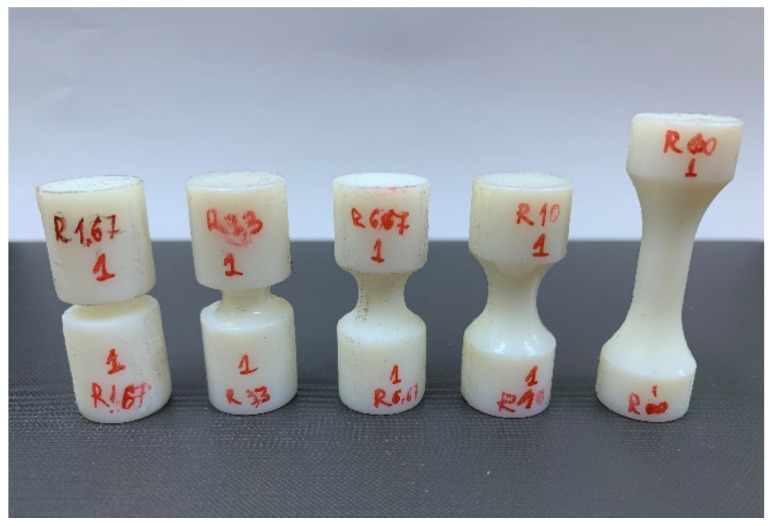
Cylindrical specimens used in upsetting tests.

Four values for the notch radii were chosen (1.67 mm, 3.33 mm, 6.67 mm and 10 mm), corresponding to theoretical values of the stress triaxiality that vary between −0.333 (un-notched specimens) and −1.12. The specimens were manufactured through photopolymerization, using the PolyJet technology and the same manufacturing parameters as the flat specimens (Figure 7).

**Figure 8:**
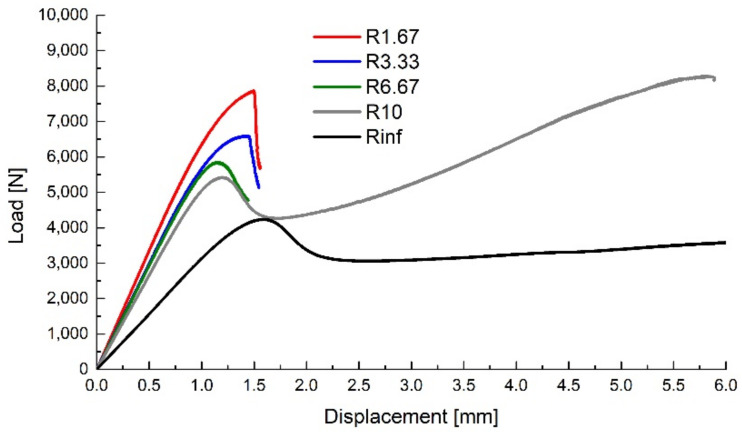
Upsetting test results.

The upsetting tests were performed on a 15 kN LFV-L Multipurpose Dynamic & Fatigue System manufactured by walter + bai (Löhningen, Switzerland) at ambient temperature using a 1 mm min^−1^ crosshead travel speed. The resulting force–displacement curves are presented in Figure 8, depicting representative curves for each specimen type.

**Figure 9:**
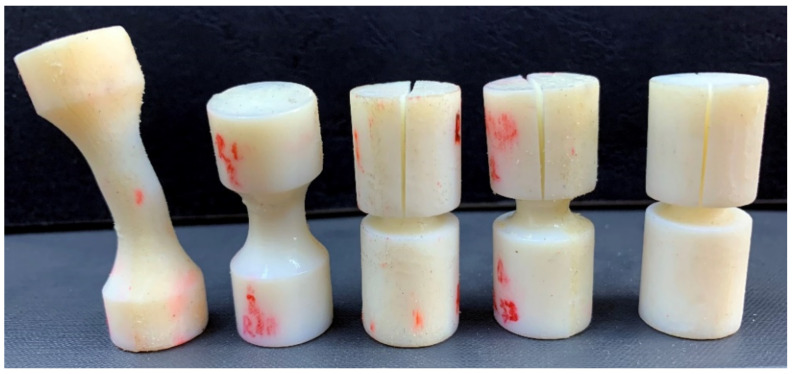
Specimen condition after the compressive tests.

During the tests, it was observed that the un-notched specimens and the specimens with a 10 mm fillet radius failed through buckling, while the rest of the specimens failed through a crack propagation that was initiated in the critical region (the outer surface of the specimen in the minimal radius region), as shown in Figure 9.

Numerical analyses were performed on axial-symmetric specimens that had the geometry identical to the revolved surface of the models used for the 3D printed specimens (the un-notched and the 10 mm notch radius specimens were omitted from the analyses, as no relevant data could be gathered). The analyses used the same material model and similar boundary conditions (fixed bottom surface and a displacement on the top surface, corresponding to the experimental values recorded at specimen damage initiation) to those used in the flat specimen simulations.

**Figure 10:**
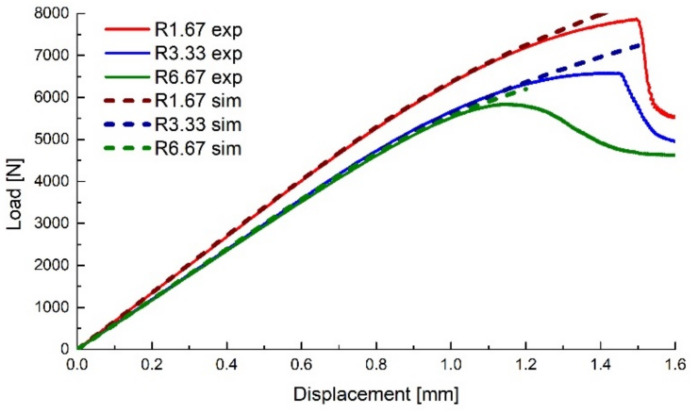
Experimental data compared with numerical results for the upsetting tests.

The displacement of the top surface and the y-axis reaction of the bottom surface were recorded as history outputs, the comparison between the experimental and numerical force–displacement curves being presented in Figure 10. A good correlation between the experimental and numerical sets of data can be observed until a given displacement for each specimen type, where the experimental values show an apparent softening, followed by a relatively rapid drop (more evident for the specimens with lower notch radii). As the sudden drop in the force values is caused by the crack propagation, the apparent softening of the material was considered to be caused by the damage evolution (void growth) in the critical region, and the separation point between the experimental and numerical data values (offset of 1% load) was considered the damage initiation point (void nucleation) for each type of specimen.

**Figure 11:**
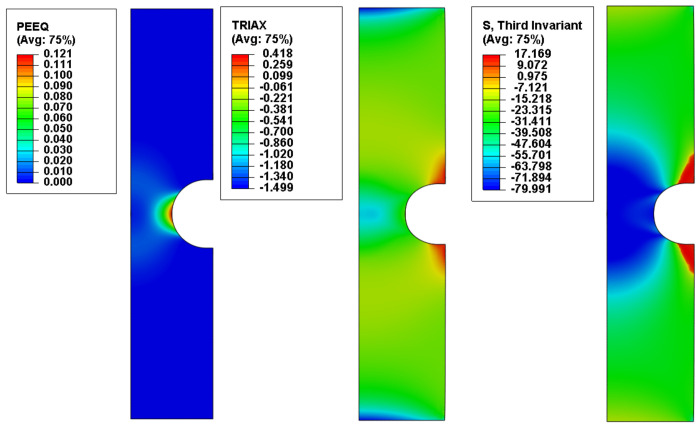
Equivalent plastic strain, stress triaxiality and normalized third invariant distribution for the upsetting tests specimens of 3.33 mm radius.

**Figure 12:**
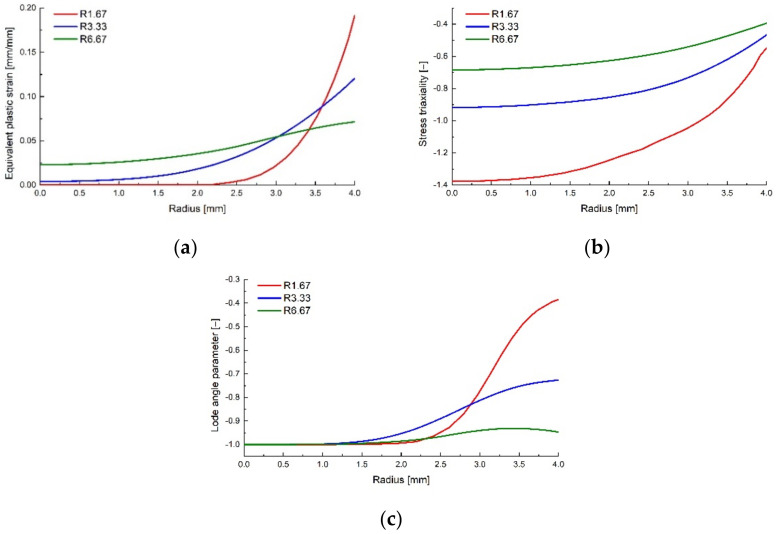
Equivalent plastic strain (**a**), stress triality (**b**) and Lode angle parameter (**c**) variation with the radius for the cylindrical specimens.

The von Mises equivalent stress $\sigma_{Mises}$, the stress triaxiality η, the normalized third invariant of the deviatoric stress tensor ρ and the equivalent plastic strain ${\bar{\varepsilon }}^{pl}$ were recorded as field output variables. Their variation from the middle of the specimen to the notch tip were recorded (Figure 11) and plotted (Figure 12) at the corresponding displacement where the damage initiation occurred.

The corresponding values of the equivalent critical plastic strain, stress triaxiality and Lode angle parameter are presented in Table 2.

**Figure 13:**
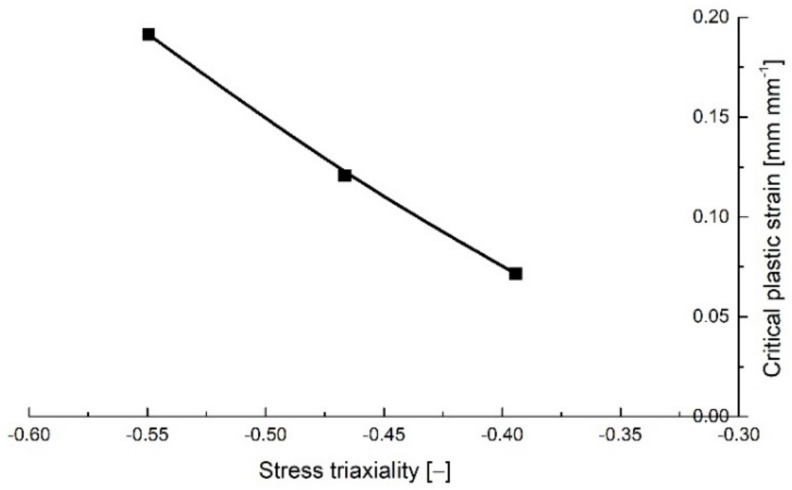
Critical plastic strain variation with stress triaxiality for the notched cylindrical specimens.

The variation of the critical equivalent plastic strain with the stress triaxiality for the round specimens is presented in Figure 13. 

## 4. Arcan Tests

**Figure 14:**
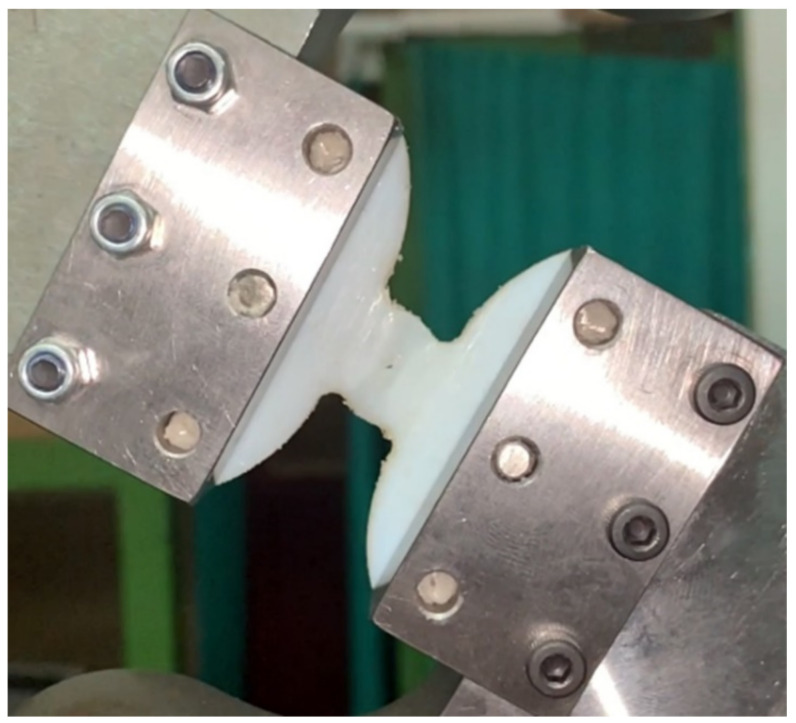
Void occurrence during Arcan tests.

The Arcan tests were described in detail in [22]. The experimental procedures were performed on rapid prototyped butterfly-shaped specimens, the design being based on the geometry proposed by Bai and Wierzbicki [10], so that the critical region was located in the middle of the specimen, as shown in Figure 14 [22].

Five orientations of the Arcan device were used: 0°, 15°, 30°, 45° and 90°providing different states of stress triaxiality, from pure shear (η=0,  ξ=0) to uniaxial tension (η=0.33,  ξ=1). The tests were performed on a 5 kN Zwick universal testing machine at mm mm^−1^ crosshead travel speed at room temperature.

**Figure 15:**
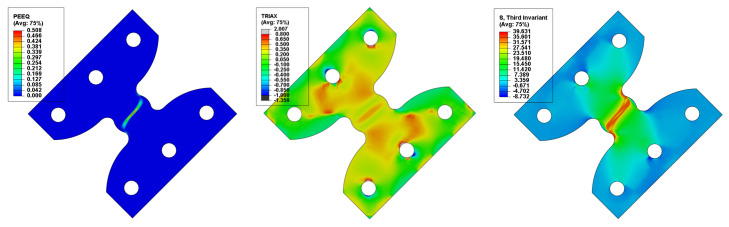
Equivalent plastic strain, stress triaxiality and third invariant distribution for the butterfly specimen oriented at 45°.

The numerical analyses (detailed in [22]) were performed using the same material model as the previous loading scenarios. Multi-point constraints were used to link the pin holes from each side of the specimens to control points. As boundary conditions, one control point was fixed and the other was assigned with a displacement, identical to the travel at fail observed in the experimental procedures. As field output, the von Mises equivalent stress $\sigma_{Mises}$, the stress triaxiality η, the normalized third invariant of the deviatoric stress tensor ρ and the equivalent plastic strain ${\bar{\varepsilon }}^{pl}$ were recorded (Figure 15), the values being probed for the centre-most element of the model.

**Figure 16:**
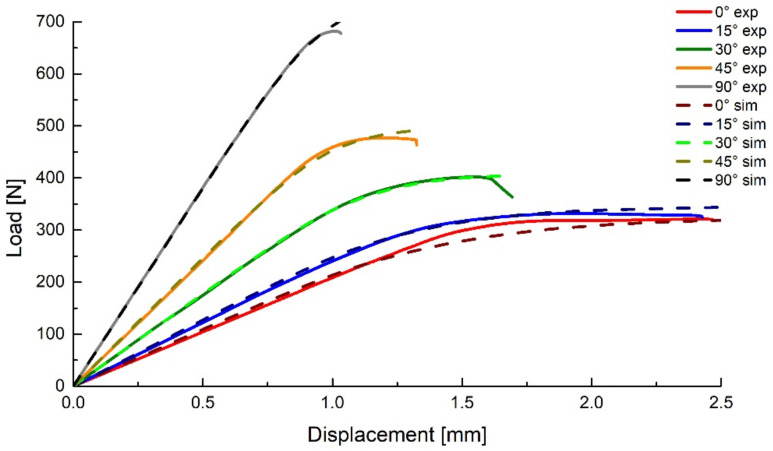
Experimental and numerical results for the Arcan tests.

The resulting force–displacement curves are presented in Figure 16, compared with representative curves form the experimental data.

As with the upsetting tests, the damage initiation was considered to occur at the displacement corresponding to the point where the numerical and experimental curves diverge. The equivalent critical plastic strain, stress triaxiality and Lode angle parameter for each loading scenario are presented in Table 3.

**Figure 17:**
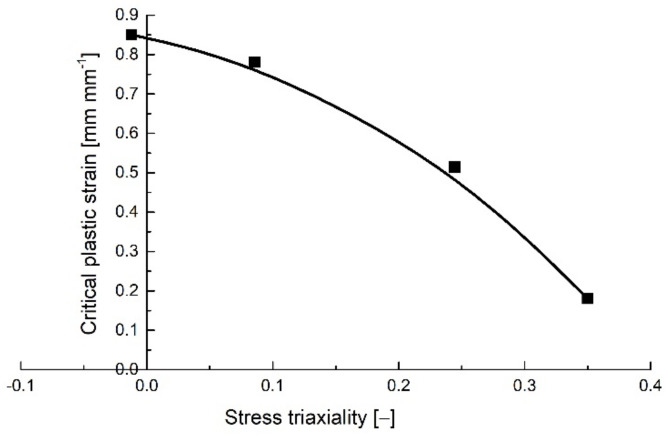
Variation of the critical equivalent plastic strain with stress triaxiality for the Arcan tests.

The variation of the critical equivalent plastic strain with stress triaxiality is presented in Figure 17.

## 5. Discussions and Conclusions

**Figure 18:**
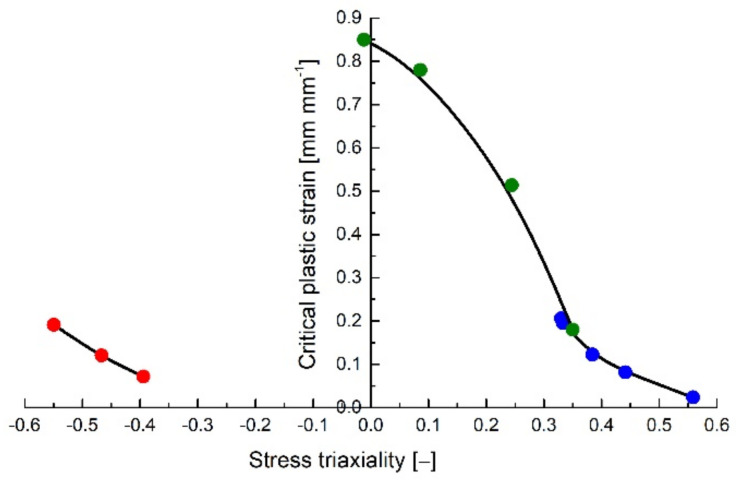
Variation of the critical plastic strain with stress triaxialities for all the investigated types of loadings.

With the help of the proposed experimental plan, the plastic strain at failure was determined for various states of stress for the investigated ABS compound. The variation of the critical plastic strain was determined for both positive stress triaxiality values (tensile tests on notched specimens determined stress triaxialities that vary between 0.333 and 0.55 while the Arcan tests between 0 and 0.35) and negative stress triaxiality values (between −0.55 and −0.4 for the upsetting tests), as seen in Figure 18.

The obtained results show that, for values of η > 0.333, the same exponential variation of the critical plastic strain with stress triaxiality observed in metals [10,12] and described by the Johnson–Cook damage model [7] was witnessed for the investigated polymer.

However, the trendlines of the failure locus of the PolyJet ABS compound differ from those obtained for metallic materials for stress triaxiality values between η=0 and η = 0.333 (predominantly shear loadings) [10,12,24]. The highest values for the critical plastic strains in the case of metals were recorded for pure tensile loadings, while the case of pure shear determines a local minimum. In contrast, for the investigated material, the highest critical plastic strain values were obtained for pure shear. 

The current experimental programme, coupled with the corresponding numerical analyses can be considered a foundation for a benchmark protocol used in determining the failure locus of polymeric materials. The damage model considered in this work (the critical plastic strain varies with the stress triaxiality and the Lode angle parameter) is implemented in commercial finite element analysis software (such as Abaqus, LS-Dyna or Pam-Crash). With adequate calibration, the model could be successfully used for predicting the failure of complex-shaped plastic components for various types of loading scenarios (that can determine a large variety of stress states), which can prove to be a very useful tool in product shape optimization and validation for companies that design and manufacture such products. 

Future work will focus on the experimental determination of the failure locus for other polymeric compounds, in order to see if the shape of the failure locus is specific for this class of materials, or if it is a particularity of the investigated ABS compound. In addition, other types of geometries and tests will be investigated, in order to determine critical plastic strain values for stress triaxialities that were not covered with the current experimental plan (namely, values between η = 0.4 and η = 0.

## Figures and Tables

**Table 1:** Equivalent critical plastic strain and Lode angle parameter variation with stress triaxiality for the notched flat specimens.

Equivalent Critical Plastic Strain [mm mm^−1^]		Stress Triaxiality [−]		Lode Angle Parameter [−]	
0.024	0.559	0.128	0.024	0.559	0.128
0.081	0.441	0.814	0.081	0.441	0.814
0.122	0.384	0.999	0.122	0.384	0.999
0.196	0.341	0.999	0.196	0.341	0.999
0.206	0.333	1	0.206	0.333	1

**Table 2:** Equivalent critical plastic strain and Lode angle parameter variation with stress triaxiality for the notched cylindrical specimens.

Equivalent Critical Plastic Strain [mm mm^−1^]	Stress Triaxiality [−]	Lode Angle Parameter [−]
0.1913	−0.549	−0.385
0.1209	−0.466	−0.726
0.0715	−0.394	−0.947

**Table 3:** Equivalent critical plastic strain and Lode angle parameter variation with stress triaxiality for the Arcan tests.

Equivalent Critical Plastic Strain [mm mm^−1^]	Stress Triaxiality [−]	Lode Angle Parameter [−]
0.18	0.349	1
0.513	0.244	0.944
0.78	0.086	0.476
0.85	−0.012	0

## Data Availability

The data supporting reported results can be provided by request.

## List of Symbols

a	Radius of the minimal cross-section of the cylindrical specimens
${\bar{\varepsilon }}^{pl}$	Equivalent plastic strain
$\varepsilon_{ij}^{pl}$	Plastic strain tensor components
${\bar{\dot{\varepsilon }}}^{pl}$	Equivalent plastic strain rate
${\dot{\varepsilon }}_{ij}^{pl}$	Plastic strain rate tensor components
${\bar{\varepsilon }}_{cr}^{pl}$	Critical equivalent plastic strain
η	Stress triaxiality
$I_{1}$	First invariant of the stress tensor
$J_{2}$	Second invariant of the deviatoric stress tensor
$J_{3}$	Third invariant of the deviatoric stress tensor
Θ	Lode angle
ξ	Lode angle parameter
r	Notch radius of the cylindrical specimens
$R_{1,2}$	Notch radii of the flat specimens
ρ	Normalized third invariant of the deviatoric stress tensor
σ	Stress tensor
σ′	Deviatoric stress tensor
$\sigma_{i}$	Principal stresses
$\bar{\sigma }$	Equivalent stress
$\sigma_{Mises}$	Von Mises equivalent stress
t	Cross-section width of the flat specimens
